# Testosterone/Epitestosterone Ratios—Further Hints to Explain Hyperandrogenemia in Children with Autism

**DOI:** 10.3390/diseases9010013

**Published:** 2021-02-01

**Authors:** Benedikt Gasser, Johann Kurz, Markus Mohaupt

**Affiliations:** 1 Department für Sport, Bewegung und Gesundheit, Universität Basel, 4052 Basel, Switzerland; 2 Intersci Research Association, Karl Morre Gasse 10, 8430 Leibnitz, Austria; john.kurz@a1.net; 3 Teaching Hospital Internal Medicine, Lindenhofgruppe, 3006 Berne, Switzerland; markus.mohaupt@lindenhofgruppe.ch

**Keywords:** steroid metabolism, gas chromatography-mass spectrometry, urine

## Abstract

Background: Epitestosterone [E] has for a long time been considered as a biologically inactive androgen. However, recently a distinct antiandrogenic activity of this naturally occurring endogenous epimer of Testosterone has been demonstrated. Especially the ratios of testosterone/epitestosterone (T/E) seem to be key as inhibition of epitestosterone on androgen activity was postulated. As in autism, a higher androgen activity was implied. We, therefore, suggested higher levels of T/E ratios of children with autism versus children with typical development. Methods: Urine probes of 22 girls with autism (BMI 18.7 ± 4.3; average age 12.3 ± 3.8 years) and a sample of 51 controls (BMI 17.0 ± 2.6; average age 11.9 ± 4 years), as well as 61 boys with autism (BMI 17.04 ± 2. average age 11.9 ± 2.5 years) and 61 control boys (BMI 17.0 ± 2.6; average age 11.1 ± 3.0 years), were analyzed with gas chromatography mass spectrometry. RESULTS: The average T/E ratio of all boys with autism was 2.5 ± 1.8 versus 2.4 ± 1.3 in boys with typical development, respectively. No significant difference between boys with autism versus boys with typical development could be detected (*p* = 0.977). In girls with autism, the average T/E ratio was 1.4 ± 0.9 versus 2.0 ± 1.4 in girls with typical development, whereby a significant difference could be detected (*p* = 0.0285). Further, polynomial analysis of the third degree were conducted, showing a dependence from age with reasonable coefficients of determination (0.075 < R^2^ < 0.22, all samples). Discussion: As encompassing steroid hormone analysis are expensive and work-intensive, we hoped to find an easily applicable biomarker to support diagnostics in autism. However, as a relatively small sample of only 22 girls with autism were analyzed and menstrual cycle and pubertal status were only partly controllable through the matching of BMI and age, the question arises if it was an incidental finding. Nevertheless, one suggestion might be that epitestosterone has the effect of a competitive inhibition on the androgen receptor, which would probably help to explain the higher prevalence of autism in boys as compared to girls. Presumably, as no significant difference was detected in boys, this effect might not be as relevant from a steroid hormone perspective, and other effects such as altered 17/20-hydroxylase activity as previously shown in boys and girls with autism seem to have more relevance. Analysis of larger samples, including plenty of metabolites and enzymatic cascades, as well as the role of backdoor pathway activity of androgen synthesis of girls with autism, are demanded in order to validate current findings of altered steroid hormones in autism.

## 1. Introduction

Autism spectrum disorder is uniformly defined as an individual’s impaired social interaction and communication deficits, including repetitive and restricted interests and behaviors. This definition is symptomatic and behavioral, yet not causative [1]. Several times an association between higher androgen levels and autism was performed. However, the focus was mainly on well-known androgens such as testosterone or dihydrotestosterone, other metabolites were not in the core of interest [2,3,4,5,6,7,8,9,10,11,12,13,14,15,16]. Epitestosterone is a natural hormone mainly produced in testes (the adrenal contribution is relatively modest), whereby on a functional level, a distinct antiandrogenic activity has been demonstrated [17,18,19]. Clear age dependence of epitestosterone plasma concentration during puberty was shown, whereby epitestosterone and testosterone showed an increase followed by a decrease during this stage of development [14,18,19,20,21]. Nevertheless, neither the biosynthetic pathways nor the site of its formation of epitestosterone in man has been unequivocally confirmed to date [18]. It apparently parallels the formation of testosterone, but on the other hand, its concentration is not influenced by exogenous administration of testosterone [14,15,16,18,19,20]. Studies showed that a complex action consisting of competitive binding of epitestosterone to androgen receptors with inhibition of testosterone biosynthesis could be demonstrated in rat, mice, and human tissues [18,19,20]. It can be presumed that epitestosterone can contribute to the regulation of androgen-dependent events [18,19,20,22]. In vitro experiments showed that the overall anti-androgenicity of epitestosterone participates true antiandrogenic action due to the binding to androgen receptors, strong 5 alpha-reductase inhibiting activity, as well as a weak anti-gonadotropic activity [18,19,20]. To sum up, all these lines of evidence indicate that epitestosterone may modulate the production, disposition, and activity of neurotransmitters, androgens, and other steroid hormones. Focusing back on alterations of androgens in autistic disorder epitestosterone might yield to hyperserotonemia influencing tryptophan metabolism [23,24,25], impairment of neurotransmitter systems [26,27,28,29,30], slower cortisol response during ACTH stimulation [31,32], higher ACTH levels [33], higher fetal testosterone [4], high DHEA [34], increased plasma oxalate levels [35], reduced pyridoxal kinase activity [27], which all might relate back to androgen dysregulation potentially influenced by epitestosterone in individuals with autism. Given that epitestosterone appears to modulate the effects of testosterone, the testosterone to epitestosterone (T/E) ratio might be the discriminating factor between individuals with autism versus those with typical development. This yields directly to the aim of the study to analyze the testosterone to epitestosterone ratios in affected children with autism versus children with typical development. As a hypothesis with potential falsification, it shall be stated that there is no difference in T/E ratios between boys and girls with autism versus typical development [2,3,36].

## 2. Material and Methods

### 2.1. Participants

A sample of 22 girls with autism (BMI 18.7 ± 4.3; average age 12.3 ± 3.8 years) and a sample of 51 girls with typical development (TD) (BMI 17.0 ± 2.6; average age 11.9 ± 4 years) was analyzed. Further, a sample of 61 boys with autism (BMI 17.04 ± 2. average age 11.9 ± 2.5 years) and of 61 control boys (BMI 17.0 ± 2.6; average age 11.1 ± 3.0 years). No significant difference for BMI or average age could be detected between the groups of affected and unaffected children.

### 2.2. Study Design

Children with autism and controls were recruited from the area of Leipzig (Austria). Enrolment took place from the mid-2009 to mid-2012 [2]. All participants were Caucasians. Participants were excluded if they had a history of liver diseases, renal or endocrine disorder, a current infection, or fever [2]. Intellectual disability or behavioral disorder were exclusion criteria only for the control group but were allowed as comorbid conditions in the group with autism, whereby one girl had to be categorized as intellectually disabled [2]. The diagnosis was given in the first years of the children’s lives according to the diagnostic criteria of the DSM-IV (Diagnostic and Statistical Manual of Mental Disorders from The American Psychiatric Association) and was cross-checked by experienced clinicians (i.e., medical doctors and/or psychologists) during enrolment of the study [37]. At the time of urine probe taking in all subjects, at least for a period of 3 months, no pharmacological intervention was made [2]. 

### 2.3. Methods

Analysis of urinary steroids was conducted via gas chromatography-mass spectrometry. Urine samples were taken in the morning after breakfast (the first urine of the day, not later than 9 a.m.) [2]. Urine sample preparation comprised pre-extraction, enzymatic hydrolysis, extraction from the hydrolysis mixture, derivatization, and gel filtration [2]. The recovery standard was prepared by adding 2.5 µg of medroxyprogesterone to 1.5 mL of urine [2]. The sample was extracted on a Sep-Pak C18 column (Waters Corp., Milford, MA, USA), dried, reconstituted in a 0.1 M acetate buffer, pH 4.6, and hydrolyzed with a powdered Helix pomatia enzyme (12.5 mg; Sigma Chemical Co., St. Louis, MO, USA) and 12.5 µL of β-glucuronidase/arylsulfatase liquid enzyme (Roche Diagnostics, Rotkreuz, Switzerland) [2]. The resulting free steroids were extracted on a Sep-Pak C18 cartridge. A mixture of internal standards (2.5 µg each of 5α-androstane-3α, 17α-diol, stigmasterol, and cholesterol butyrate, and 0.15 µg of 3β5β-tetrahydroaldosterone) was added to this extract, and the sample was derivatized to form the methyloxime-trimethylsilylethers [2]. Analyses were performed on a Hewlett Packard gas chromatograph 6890 (Hewlett Packard, Palo Alto, CA, USA) with a mass selective detector 5973 by selective ion monitoring (SIM) [2]. One characteristic ion was chosen for each compound measured. The derivatized samples were analyzed during a temperature-programmed run (210–265 °C) over a 35 min period. The calibration standard consisted of a steroid mixture containing known quantities of all the steroid metabolites to be measured [2]. Responses and retention times were recorded regularly. In each case, the ion peak was quantified against the internal stigmasterol standard, whereby all procedures were performed as previously described by us and others [2,38,39,40].

### 2.4. Statistical Analysis

For all subsamples (boys with autism, boys with typical development, girls with autism, girls with typical development), the T/E ratios mean and standard deviation were calculated. Kolmogorov–Smirnov tests were conducted to test for normal distribution, whereby the hypothesis of the normal distribution of T/E ratios could not be rejected for all subsamples. Differences were analyzed between samples with two-tailed heteroscedastic *t*-tests. To correct for multiple comparison, a Bonferroni correction was performed. Calculations were performed with GraphPad Prism (GraphPad Software, Inc., La Jolla, CA, USA) and Microsoft Excel (Microsoft Inc., Redmond, WA, USA).

## 3. Results

The average T/E ratio of all boys with autism was 2.51 ± 1.8 versus 2.43 ± 1.3 boys with typical development. No significant difference between boys with autism versus typical development could be detected (*p* = 0.977). In girls with autism, the average T/E ratio was 1.37 ± 0.9 versus 2.03 ± 1.4 in girls with typical development, whereby a significant difference could be detected (*p* = 0.0285).

**Figure 1:**
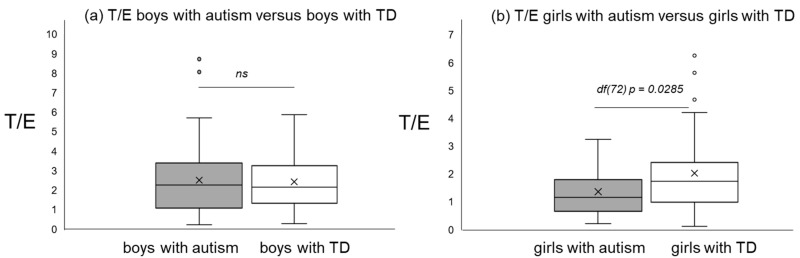
T/E ratios of boys with autism versus typical development (**a**) and T/E ratios of girls with autism versus typical development (**b**).

**Figure 2:**
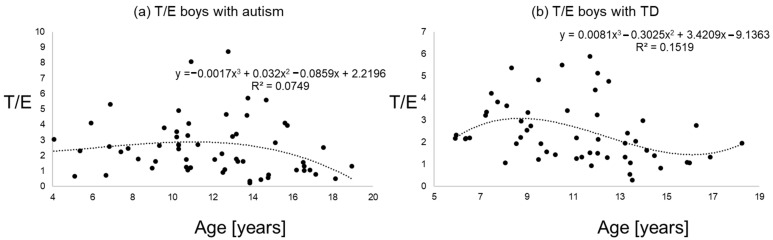
T/E ratios of boys with autism (**a**) versus typical development (**b**) the line shows a polynomial interpolation of third degree.

**Figure 3:**
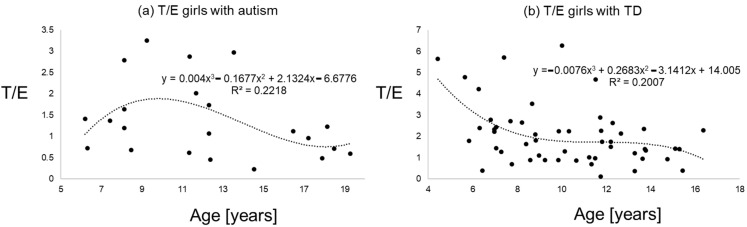
T/E ratios of girls with autism (**a**) versus typical development (**b**) the line shows a polynomial interpolation of third degree.

Figure 1, Figure 2 and Figure 3 give an impression of the relationship of age versus T/E ratios for the four subsamples {a) boys with autism versus boys with TD and (b) girls with autism versus girls with TD. In all calculated polynomial analysis coefficient of determination (R^2^) was between 0.075 and 0.22.

## 4. Discussion

The aim of this study was to analyze testosterone/epitestosterone ratios in affected children with autism versus typical development. We hoped to find an easily applicable biomarker to support diagnostics in autism. As encompassing steroid hormone analysis are expensive and work-intensive, and for methodological reasons, some further variance results from kidney function, we here focused our analyzes simply on ratios of testosterone/epitestosterone levels. However, no differences could be detected in boys with autism versus boys with typical development. Nevertheless, in girls with autism a decrease of T/E ratios was detected, suggesting lower concentrations of testosterone or higher concentrations of epitestosterone. Reasons for these different findings between sexes remain on the first glue speculative. As a relatively small sample of only 22 girls with autism were analyzed, and menstrual cycle and pubertal status were only partly controlled through the matching of BMI and age, the question arises if it was an incidental finding. In general, evidence for girls with autism is small compared to boys and in our analyses as well, we only had around a third of the sample size analyzed compared to boys. On the other hand, do the results indicate some protective effect from epitestosterone? Might it be possible that, for example, a competitive binding on androgen receptors exists, yielding to an antiandrogenic activity? This might explain, for example, the around four times higher prevalence of autism in boys versus girls [1,4]. Besides the relatively small samples analyzed, the onset of puberty remains critical as it influences the levels of epitestosterone and testosterone. The likely onset of puberty (Standard deviations of the sample were around four years and, therefore, encompassing a relatively large time span) in some participants of this study confounds the interpretation of the results in consequence. Furthermore, as we were able to show in a previous work, the increased testosterone concentrations in girls and boys with autism, there is likely an increase of epitestosterone [2,3]. Interestingly, Gustafsson et al. (1976) found that the female rat brain more rapidly metabolizes testosterone to so-called ‘inactive compounds’, including epitestosterone [41]. The metabolism and binding of testosterone in the male and female rat brain has been studied in an attempt to find an explanation for the relative androgen unresponsiveness characterizing the female hypothalamo–pituitary axis involved in regulation of steroid metabolism [41]. One possible explanation for the androgen unresponsiveness of female rats is, therefore, the faster metabolism of testosterone to inactive compounds in the female brain [41]. Experiments both in vivo and in vitro showed the presence of high affinity, low-capacity binding sites for testosterone in the male pituitary, pineal gland, and hypothalamus [41]. On the basis of these results, it was suggested that the androgen unresponsiveness of female rats referred to the above-related absence of receptor protein for androgens in the female rat brain [41]. In conclusion, the relative androgen unresponsiveness of the female hypothalamo–pituitary axis is probably explained by the absence of receptor proteins for androgens in the female hypothalamus and pituitary [41]. It was shown that concentrations of epitestosterone were age-dependent and, at least in prepubertal boys and girls, epitestosterone reached or even exceeded the concentrations of testosterone, thus supporting its role as an endogenous antiandrogen [16]. The detected pattern of a peak around nine to eleven years, probably due to starting puberty, was in line with previous findings [16,17,18,19,20]. The values of ratios in boys with autism versus controls were around 2.5, in line with findings from others [14,15,16]. Still, reasons for the most obvious finding of lower T/E ratios in girls with autism remain speculative. Different T/E ratios are influenced by UGT2B17 polymorphism yielding to some further unexplainable variance [14,42,43]. Interestingly, when the UGT2B17 genotypes were compared with urinary testosterone levels, all of the individuals of the UGT2B17 homozygous deletion/deletion genotype had no or negligible amounts of urinary testosterone whereby UGT2B17 polymorphism was strongly associated with testosterone excretion and depended on ethnicity and sex [42,43].

To sum up, a principle hyperandrogenemia in boys with autism seems now secured, albeit evidence on girls remains sparse [1,2,3,4,5,6,7,8,9,10,11,12,13]. In consequence, the pharmacological role of epitestosterone in autism remains unclear [18,19,20]. One premise might be that it has the effect of a competitive inhibition on the androgen receptor. However, in contrast to testosterone, no or a smaller activation takes place, yielding in total to an antiandrogenic effect. However, as no significant difference was detected in boys, this effect might not be as relevant as other effects such as increased 17/20-hydroxylase activity as already stated [2,3,13]. This opens up a wide field of speculation remaining unclarity concerning the specific mechanism of dysregulations of steroid hormones in autistic diseases, whereby in several future aspects such as enzymatic activity, measurement in plasma versus urine, and the role of a backdoor pathway of androgen synthesis have to be discussed [2,3,13]. Furthermore, future studies may benefit from examining cohorts strictly before and after puberty with adequate assessment of the respective state (for example, with Tanner stages) in order to receive more information concerning the dynamics over the time of puberty of steroid hormones in children with autism compared with neurotypical controls. As the severity of autism might be clearly related to steroid hormone levels, further hints, for example, from a protective effect of two X chromosomes would result from the parallel analysis of the severity of autism and steroid hormone levels.

## Data Availability

Data sharing not applicable, as informed consent was only received for own usage of data from participants.
