# Expression of Concern: Gasser et al. Testosterone/Epitestosterone Ratios—Further Hints to Explain Hyperandrogenemia in Children with Autism. *Diseases* 2021, *9*, 13

**DOI:** 10.3390/diseases13030065

**Published:** 2025-02-21

**Authors:** Diseases Editorial Office

**Affiliations:** MDPI, St. Alban-Anlage 66, 4052 Basel, Switzerland; diseases@mdpi.com

With this notice, the *Diseases* Editorial Office states their awareness of the concerns regarding potential scientific errors and an authorship dispute relating to this publication [1]. The corresponding author and the relevant institution have been contacted for further clarification on this matter. In order to maintain the highest scientific standards, the Editorial Office has decided to issue this expression of concern while an investigation is being conducted.

Once this process has been completed, the Editorial Office will provide an update on this situation and announce any post-publication modification deemed to be necessary, as per MDPI’s Update to Published Papers policy.
